# Effects of climate variability on the spatio-temporal distribution of Dengue in Valle del Cauca, Colombia, from 2001 to 2019

**DOI:** 10.1371/journal.pone.0311607

**Published:** 2024-10-08

**Authors:** Delia Ortega-Lenis, David Arango-Londoño, Freddy Hernández, Paula Moraga

**Affiliations:** 1 School of Statistics, Faculty of Sciences, Universidad Nacional de Colombia, Medellín, Colombia; 2 Department of Public Health and Epidemiology, Pontificia Universidad Javeriana, Cali, Colombia; 3 Department of Mathematics, Pontificia Universidad Javeriana, Cali, Colombia; 4 Computer, Electrical and Mathematical Sciences and Engineering Division, King Abdullah University of Science and Technology (KAUST), Thuwal, Saudi Arabia; Universidad San Francisco de Quito, ECUADOR

## Abstract

Dengue is a vector-borne disease that has increased over the past two decades, becoming a global public health emergency. The transmission of dengue is contingent upon various factors, among which climate variability plays a significant role. However, there remains substantial uncertainty regarding the underlying mechanisms. This study aims to investigate the spatial and temporal patterns of dengue risk and to quantify the associated risk factors in Valle del Cauca, Colombia, from 2001 to 2019. To achieve this, a spatio-temporal Bayesian hierarchical model was developed, integrating delayed and non-linear effects of climate variables, socio-economic factors, along with spatio-temporal random effects to account for unexplained variability. The results indicate that average temperature is positively associated with dengue risk 0-2 months later, showing a 35% increase in the risk. Similarly, high precipitation levels lead to increased risk approximately 2-3 months later, while relative humidity showed a constant risk within a 6 months-lag. These findings could be valuable for local health authorities interested in developing early warning systems to predict future risks in advance.

## Introduction

Dengue is a widespread vector-borne disease caused by the dengue virus, which is primarily found in tropical and subtropical regions, particularly in urban and semiurban areas. The incidence of dengue has risen significantly in the last two decades, becoming a global public health concern due to its health and economic impacts [1]. In 2023, it was estimated that half of the world’s population was at risk of dengue, with 100 to 400 million infections occurring annually. In the Americas, there were over four million reported cases in 2023, with an incidence rate of 456 cases per 100,000 inhabitants, marking a 115% increase compared to the average of the previous five years [2].

Dengue viruses are transmitted to people through the bite of an infected *Aedes* species (*Aedes aegypti* or *Aedes albopictus*) mosquito. The spread of these mosquitoes is influenced by several factors such as social, economic and climatic conditions, particularly impacting vulnerable populations in low-income urban areas [3]. Numerous studies have demonstrated the relationship between vector-borne diseases and socioeconomic factors, identifying key determinants such as the lack of access to water, which results in water storage practices, along with inadequate sanitation services, poor sewage and waste management that creates ideal breeding grounds for *Aedes aegypti* mosquitoes. Additionally, education, income, age, access to care are known to strongly influence the susceptibility to these diseases [4–7].

Climate’s impact on health, particularly in relation to infectious diseases like dengue, has been studied by multiple authors [8, 9]. Studies found that climate change can affect pathogens, hosts, and the transmission environment of these diseases. For example, the development, behavior, and survival of the *Aedes* mosquitoes depend on temperature and humidity, while precipitation is necessary for egg laying [10]. Studies suggest that mosquito densities are highest at temperatures between 15°*C* and 32°*C* [11–13] with a non-linear relationship due to the harmful effects of very high temperatures on mosquito eggs. After a rainy season, an increase in mosquito densities is expected, but this depends on the accumulation and intensity of rainfall. Intense rainfall can actually reduce mosquito populations by washing away eggs and larvae [11, 14].

Colombia provides a suitable habitat for the primary mosquito vector of dengue, *Aedes aegypti*, which is widespread across the country. In Colombia, disability-adjusted life years (DALY) caused by dengue are between 9845.9 and 13584.2 DALY per million inhabitants in epidemic years [15]. Last year, the country reported the highest frequency of severe dengue cases in South America, with a 100% increase compared to 2022 [16]. Therefore, dengue generates a very high cost to the Colombian healthcare system and economic expenses for the population.

In dengue studies conducted in Colombia, a prominent approach involves the use of generalized linear mixed models that incorporate spatial and temporal variability using linear effects of covariates and random effects [17–19]. However, these models overlook the complexity of the relationships between dengue and the covariates utilized by assuming linear effects. This research aims to analyze the effects of climate and socio-economic factors on dengue risk in Valle del Cauca, Colombia, considering non-linear and delayed effects as well as spatial and temporal random effects to model residual variation.

## Materials and methods

### Study area

**Fig 1:**
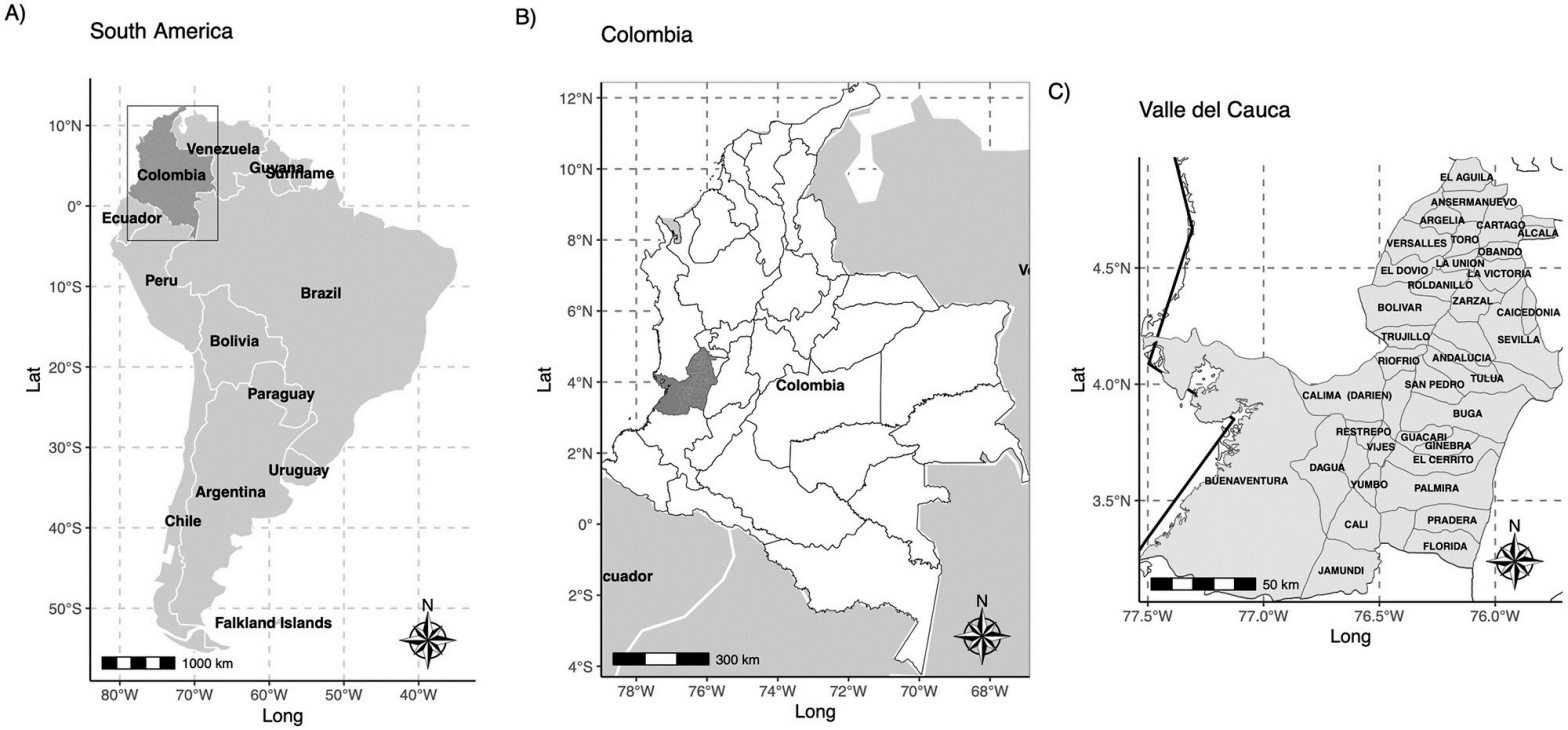
Geographical location of the study region, Valle del Cauca, Colombia. Basemap shapefile downloaded from https://rspatialdata.github.io/admin_boundaries.html.

Colombia is a country with over 52 million inhabitants, organized into 32 departments and 1104 municipalities. One of its departments, located in the southwest of the country, is the Valle del Cauca (Fig 1), with a population of 4.6 million in 42 municipalities, 5 subregions, and an area of 22,195 km^2^ (at 3.56° N, 74.30° W, and 1561m above sea level). Its territory stretches from the Pacific coast, through the Western and Central Andes. It is characterized by an intertropical climate with two dry seasons per year (December to February and June to September) and two rainy seasons (March to May and October to November). However, the Pacific subregion stands out with high precipitation, averaging around 231 rainy days per year and a brief dry season in January and February.

### Dengue and population data

Confirmed and reported dengue cases were obtained from the public health surveillance system (SIVIGILA) for each of the 42 municipalities in Valle del Cauca between January 2000 and December 2019 [20]. The database included potential and confirmed cases in the laboratory using hemogram and immunoglobulin M [IgM] tests. Dengue data were downloaded from the website of the National Institute of Health (INS) (available at https://www.ins.gov.co/buscador-eventos/Paginas/Info-Evento.aspx). To calculate monthly rates, population estimates for each municipality and year were downloaded from the National Administrative Department of Statistics (DANE) [21].

### Climate and socio-economic data

The climate variables utilized in this research included monthly average, maximum and minimum temperatures (°*C*), mean relative humidity (%), and accumulated monthly precipitation (mm). These data were sourced from The Copernicus Climate Change Service (C3S, version 1.0) [22] for the period from January 2000 to December 2019. This dataset is based on hourly ECMWF ERA5 data at the surface level, aggregated to daily time steps in the local time zone, and corrected for finer topography at a spatial resolution of 0.1°. The gridded data were further aggregated by month and municipality by calculating the mean of grid boxes within a buffer around the municipal capital, using the municipality’s area radius. Temperature indicators, such as the range of maximum and average monthly temperatures, were computed for inclusion as variables in the model. Additionally, El Niño Southern Oscillation (ENSO) obtained using monthly Niño-1.2 and Niño-3.4 data was retrieved from the National Oceanic and Atmospheric Administration (NOAA) [23]. This indicator is commonly used in prior studies which relate climate and dengue in Colombia due to its proximity to Pacific coast [12, 19].

Climate data were validated using information from Cali, the capital of the department. Specifically, data extracted from aggregated images were compared to the meteorological station data in Cali for the period 2000 to 2014. It was found that the average, maximum, and minimum temperatures in the images were lower than the actual values from the monitoring station (S1 Fig). Correlations above 65% were found between the different series, indicating a strong relationship. Linear regression models were used to adjust the temperature series, using slope values equal to 1.16°C for maximum temperature, 1.81°C for minimum temperature, and 1.44°C for average temperature (S1 Table).

Socio-economic factors were obtained from the 2018 census by DANE [21]. These included the proportion of households with water supply, the proportion of households with sewerage, the proportion of the population residing in urban areas, and the proportion of households in low socio-economic strata (stratum 1 and 2). This last indicator is an ordinal measure validated by the National Department of Planning, with values ranging from 1 to 6, where 1–2 represents low, 3–4 middle, and 5–6 high economic capacity. This classification is based on household characteristics, including construction materials, housing conditions, and immediate surroundings such as access roads and sidewalks. Each house is assigned a specific stratum, and each neighborhood is classified according to the mode [24].

### Modelling framework

A spatio-temporal Bayesian hierarchical model [25] was formulated using monthly counts of notified dengue cases for 43 municipalities in Valle del Cauca, from January 2001 to December 2019. Counts of dengue cases *y*_*st*_, *s* = 1, …, 42, *t* = 1, …, 228 were assumed the following negative binomial distribution:

$$\begin{array}{c} y_{st}|\mu_{st,}\kappa ∼NegBin\left(\mu_{st,},\kappa \right) \end{array}$$ (1)

$$\begin{array}{c} \text{log}\left(\mu_{st}\right)=\text{log}\left(p_{s\;a(t)}\right)+\text{log}\left(\rho_{st}\right). \end{array}$$ (2)

Here, *μ*_*st*_ represents the mean number of cases in each municipality *s* and month *t*, and *κ* is the scale parameter. In the model, population effects are accounted by including the logarithm of the population per 100,000 as an offset at the linear predictor scale. Thus, *μ*_*st*_ is expressed as the yearly population per 100,000 (*p*_*s a*(*t*)_, *a*(*t*) = 2001, …, 2019) multiplied by the dengue incidence rate (*ρ*_*st*_).

The dengue rate *ρ*_*st*_ was modeled using a combination of climate and socio-economic factors as well as spatial and temporal random effects. To select the optimal combination of covariates and random effects, we compared several models using the Deviance Information Criterion (DIC) [26] and Watanabe-Akaike information criterion (WAIC) [27]. We initially specified a baseline model that accounted for seasonality and interannual spatial variability at the municipality level using random effects. Subsequently, we separately added covariates from the climate and socio-economic groups to this baseline model to select the most important climatic variables and socio-economic factors. Finally, the variables from these two groups that resulted in the model with the smallest DIC and WAIC were put together and added in the model that also included the random effects.

For the final model, we simulated the posterior predictive distribution of the response variable using samples from the posterior distribution. This was done 19 x 12 times, leaving out a month per year each time [28]. Then, the posterior predicted medians were compared to the observed dengue rates. The model parameters were estimated using the integrated nested Laplace approximation (INLA) in R [29, 30].

The baseline model for the dengue incidence rate accounted for seasonality at the municipality level by including a municipality-level random effect per calendar month, and interannual spatial variability by using a year-specific effect that accounted for spatially structured and unstructured variability. Specifically,

$$\begin{array}{c} \text{log}\left(\rho_{st}\right)=\alpha +\beta_{s\;m(t)}+\phi_{s\;a(t)}+\nu_{s\;a(t)}, \end{array}$$ (3)

where *α* is the intercept, and *β*_*s m*(*t*)_ accounts for the seasonality at the municipality level and is represented with a cyclic random effect of first order for each municipality and month with no discontinuity between January in year *a*(*t*) and December in year *a*(*t*) − 1. Interannual variability and long-term trends are accounted for using an interaction between year and spatially structured (*ν*_*s a*(*t*)_) and unstructured random effects (*ϕ*_*s a*(*t*)_). This component utilized a Besag-York-Mollie (BYM) model with a conditional autoregressive model (CAR) as a prior in the structured spatial random effect, and an independent municipality-specific noise random effect [31, 32]. Here, structured spatial random effects allowed for dependency between municipalities, while unstructured random effects accommodated other unmeasured factors in the municipalities.

The baseline model was extended by including linear effects of the socio-economic variables. Additionally, distributed lag non linear models (DLNMs) were used to include possible non-linear and delayed associations between dengue incidence rate and climate variables since several studies have documented this type of relationship [17, 33]. DLNMs are based on the definition of a cross-basis obtained by the combination of two functions that account for non-linear exposure-response effects *f*(*x*), and the lag structure of the relationship *w*(*l*) [34]. Thus, the baseline model for dengue incidence rate was extended using non-linear exposure-lag functions for the climatic variables *f*.*w*(*x*, *l*). Following the model employed for dengue risk in Brazil by [17], these functions were constructed using lags betwen 0 and 6 months, and natural cubic splines for both the exposure (two equally spaced knots) and the lag dimension (with one internal knot at the percentile 50). We constructed the cross-basis functions for the DLNM component using the R package dlnm in [35]. Additionally, we compared this model with a model that included lagged linear variables using DIC and WAIC. For reproducibility, we made data and code available at https://github.com/deliaortegalenis/Dengue-Climate.

## Results

### Dengue and socio-economic variables

**Fig 2:**
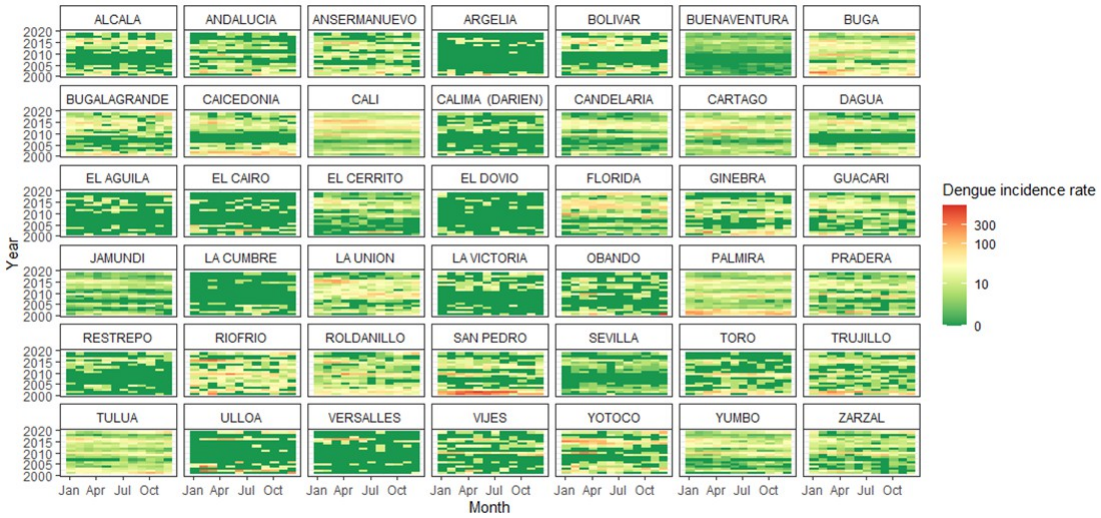
Dengue incidence rate in Valle de Cauca from 2001 to 2019.

Between 2000 and 2019, SIVIGILA registered 170,977 dengue cases, with o without warning signs which could include abdominal pain, persistent vomiting, and mucosal bleed [36], in the 42 municipalities of Valle del Cauca. Dengue rates have increased over this period, but the increase varies between municipalities. Additionally, the monthly pattern of dengue rates varies across municipalities. For example, in some cities like Cali (the capital), Andalucia, Ansermanuevo, Bolivar, Cartago, Dagua, and Riofrío, the transmission season occurs in the first months of the year. In other municipalities, high rates can occur in any month of the year, and this variation is not dependent on the geographical zone. The years with dengue epidemics were 2002, 2010, 2013, 2015, and 2016 (Fig 2).

The socio-economic variables obtained in the census of 2018 show that municipalities in the north-west and south-west of the department have the lowest percentages of access to water supply (70–80%), residents living in urban areas (20–50%), households with sewer (70–80%), and high percentages of households with low socioeconomic strata (50–70%) (S2 Fig). We use the proportion of households with the lowest socio-economic strata (1–2) as a proxy for vulnerable conditions, which is negatively correlated with the other variables. Specifically, the Pearson correlation coefficient with water supply was *r* = −0.62 (*p* < 0.05), with urban population *r* = −0.52 (*p* < 0.05) and with sewer *r* = −0.51 (*p* < 0.05). However, the correlation between proportion of households with water supply and proportion of residents living in urban areas was positive *r* = 0.61 (*p* < 0.05). This indicates that in municipalities with higher urbanization, better access to water supply is expected, but a higher population in urban areas also increases the risk of dengue.

### Climate factors

Temperature varies between municipalities in Valle del Cauca. For instance, the mean temperature in cities like Buenaventura on the Pacific coast is consistently above 29°*C* almost throughout the twelve months of the year, while in other cities like Calima, it averages around 23°*C*. Maximum temperatures peak during the dry season between June and September (S3 Fig). Precipitation follows a typical pattern, with rainy months occurring from March to May and October to November (S4 Fig). While relative humidity does not exhibit a clear temporal pattern throughout the year, it shows variability between municipalities, with values ranging between 61% to 87% (S5 Fig).

### Effects of climate and socioeconomical variables in Dengue risk

In total, we estimated 48 models resulting from the combinations of eight climate variables and four socio-economic factors, each comprising the random effects of the baseline model and various combinations of climatic and socio-economic variables. We then selected the best model based on the DIC and WAIC. Among the climate variables, the combination of precipitation, mean temperature, and relative humidity yielded the lowest DIC and WAIC when considering lagged non-linear effects. We then compared this combination with linear terms and observed that both indicators decreased with the inclusion of non-linear terms. Specifically, DIC decreased from 44,240 to 44,070, and WAIC decreased from 44,400 to 44,213. For socio-economic factors, including water supply, urban population, and low stratum improved the model fit. However, due to the high and significant correlation between these factors, we selected the second model with the lowest DIC and WAIC, resulting in the inclusion of the water supply variable.

We then estimated using the selected climate and socio-economic variables, which led to an improved model fit compared to both the baseline and the previously mentioned models (Table 1). However, since the change in DIC and especially WAIC was minimal, we opted to select the final model containing only the climate variables. We also validated the results, finding that the estimated risk for the climate variables did not change between the model with water supply and the model without it.

**Table 1:** Model adequacy results.

Model	DIC	WAIC
Base model (BM): log(*ρ*_*st*_) = *α* + *β*_*s m*(*t*)_ + *ϕ*_*s a*(*t*)_	58243.7	02792.4
BM + Mean temperature + Precipitation + Relative humidity	44070.9	44213.7
BM + Water supply + Urban population + Low stratum	44265.2	44214.8
BM + Water supply	44307.8	44466.2
BM + Mean temperature + Precipitation + Relative humidity + Water supply	44024.83	44175.8

**Fig 3:**
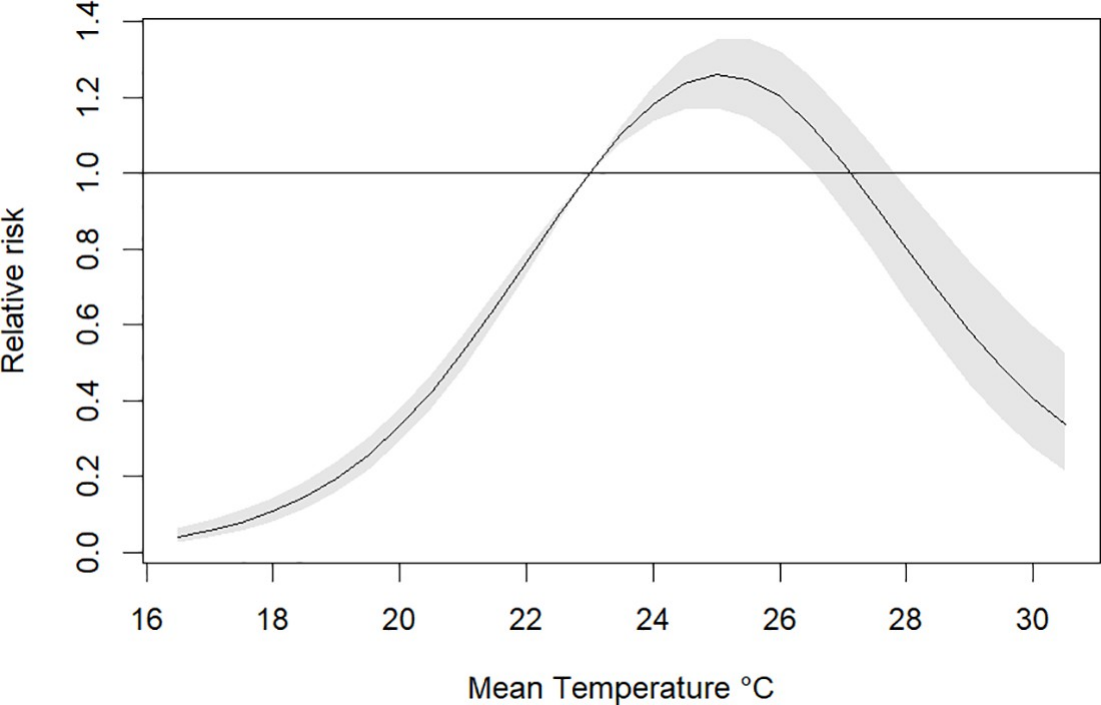
Overall exposure-response association across all lags for mean temperature.

**Fig 4:**
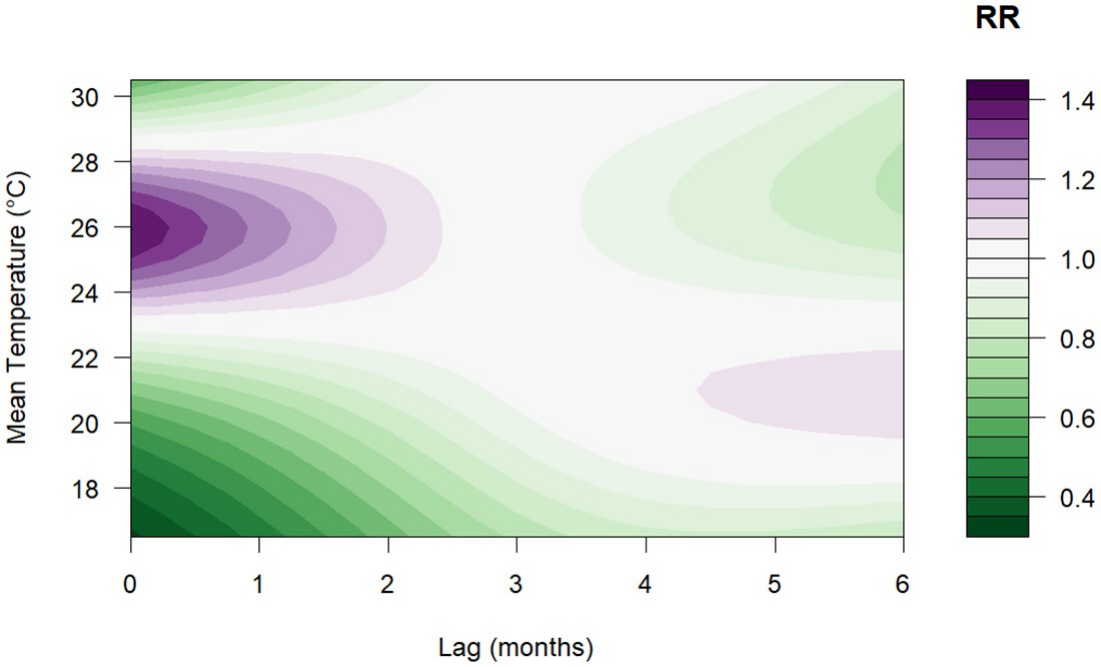
Contour plot of the exposure-lag-response association (RR) between mean temperature and risk of dengue (compared to overall mean of 22.6°*C*).

**Fig 5:**
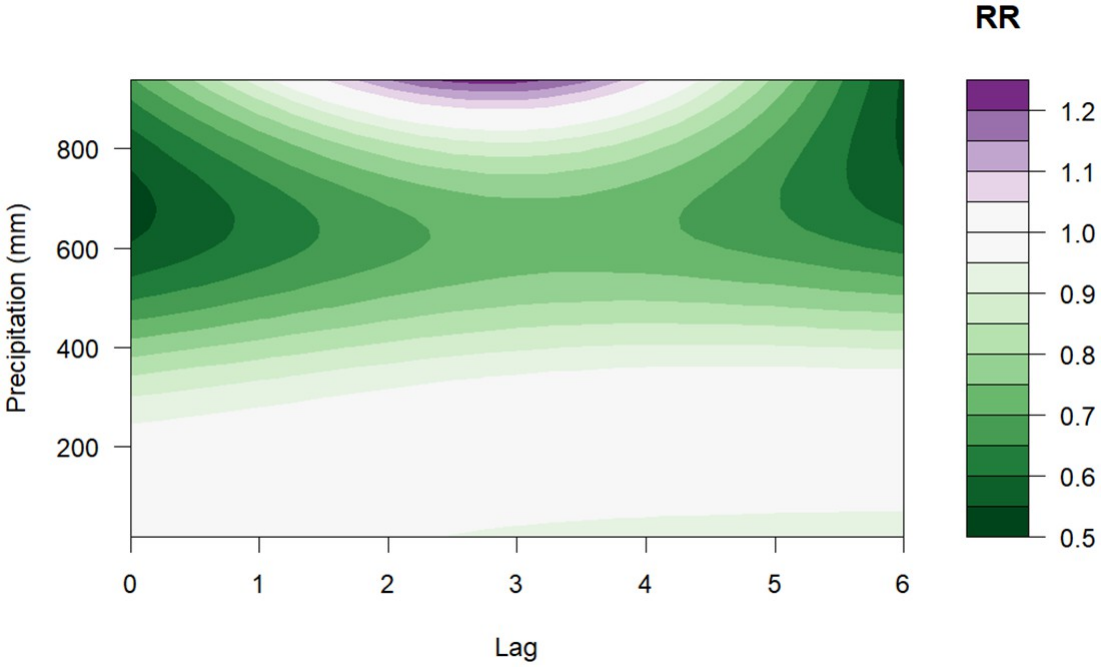
Contour plot of the exposure-lag-response association between precipitation and risk of dengue (compared with overall mean of 152.1 mm).

**Fig 6:**
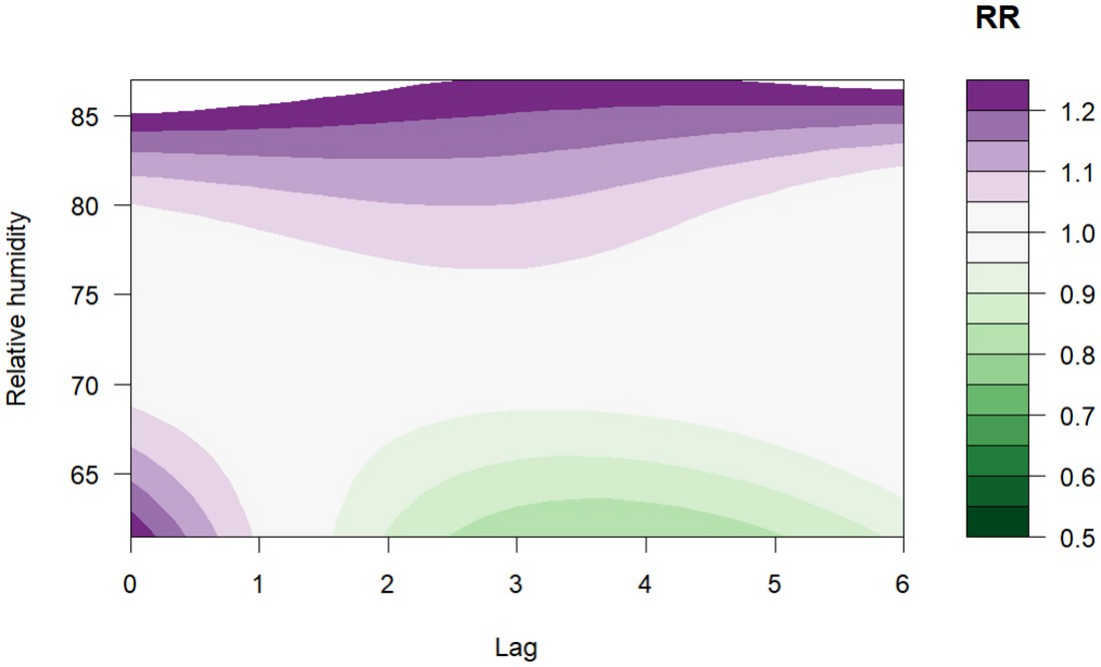
Contour plot of the exposure-lag-response association between relative humidity and dengue (compared with overall mean of 72.5%).

Fig 3 illustrates that the relative risk (RR) of dengue increases with rising mean temperatures but after 27.5°*C* the risk is below one. The highest RR of 1.40 (95% CI: 1.17–1.67) was observed at a temperature of 26°*C* with a lag between 0–2 months (Fig 4). Regarding precipitation, values above 800 mm indicated a high risk of dengue, the highest RR was 1.22 (95% CI: 0.78–1.93) for a value of 940 mm with a lag of 2–3 months, as shown in the contour plot in Fig 5. Finally, for relative humidity, the highest RR was 1.35 (95% CI: 0.69–2.64) for a value of 87% with a lag of 0 months, however, the risk is almost constant for all the lags as depicted in Fig 6.

Finally, the predicted values obtained in the out of sample posterior estimates of dengue incidences, simulated from the final model fitted in the cross-validation process that excluded one month per year at a time, shows that the predicted rates vary between municipalities where the west cities in the department have the higher rates. Epidemiological reports and other studies conducted in Colombia have identified dengue outbreaks for 2002, 2010, 2013, 2015 and 2016 [10, 15, 16]. Our findings reveal that during these years, the highest rates were reported across all municipalities (S6 Fig).

## Discussion

In this study, we employed a spatio-temporal model to analyze the lagged and non-linear effects of climate and socio-economic variables on dengue risk in Valle del Cauca, Colombia, which is the second most populated department and one of the regions with the highest number of dengue cases and deaths [16]. The findings indicate that average temperature exhibited a positive association with dengue risk 0–2 months later, showing a 40% increase in the rate. Similarly, high precipitation levels accumulated in the month lead to increased risk approximately 2–3 months later. For humidity, the effect was more immediate without lag time and remained relatively constant over the 6 months.

These results are consistent with multiple investigations where climate variables such as average temperature, precipitation, and relative humidity appear to have significant effect in explaining dengue risk [3, 5, 10, 11, 18]. In the case of average temperature, results in Colombia and in the city of Cali report positive associations with lags of up to two weeks [3, 10]. For precipitation, findings from other studies vary; in some cases, it increases the risk of dengue in vulnerable areas [37, 38], meaning its effect is mediated by socio-economic factors such as sanitary conditions and access to water supply and sewage systems, as water storage practices increase the availability of habitats for *Aedes aegypti* larvae. In other cases, precipitation shows a decrease in dengue risk since heavy rains could overflow outdoor containers and wash them away, depending on the availability of tanks inside and outside households [17]. Regarding relative humidity, the expected relationship is positive, as vector feeding is more frequent during dry periods. While many studies have evaluated this effect, some report a significant relationship of mean relative humidity and rate of dengue. In Colombia, research has shown a significant effect of this variable with a 4 week lag in one study and an 8-week lag in another [3, 5].

Regarding socio-economic variables, this study found that access to water supply improves the model fit using DIC, which is consistent with other research where is found a relationship between socio-economic inequalities and the incidence of such diseases in Colombia and Valle del Cauca [4, 39]. However, the difference in DIC and WAIC between the model with this variable and without it was minimal, and the estimated rate risk did not change, indicating that this factor does not effectively control for potential confounding effects. This may be because it does not accurately measure the percentage of households lacking continuous access to quality water, as water cuts can be frequent. A study conducted in Brazil included the variable “water cuts” for a more precise measurement of storage or use of alternative sources of water [17]. However, obtaining this information during the study period was not feasible for this research.

It should be noted that the estimated risks do not show large magnitudes, except for relative humidity. Concerning temperature, the patterns remain relatively stable due to Colombia’s absence of distint seasons, except during El Niño or La Niña events, which trigger significant fluctuations in temperature and precipitation, respectively. Although in this study the Niño index 1.2 or 3.4 did not impact the model fit, this differs from findings from other research conducted in Colombia where ONI (Oceanic Niño Index) and Niño 1.2 were considered as variables to represent the dynamics of ENSO phenomenon, and showed a strong relationship with the incidence of arboviruses such as dengue [19, 40]. Although these findings indicate that El Niño increases dengue cases in the country, these analyses were conducted at the national level and not for a specific region or department, as in this study.

Dengue incidence in Valle del Cauca has been affected by interventions implemented over the 19-year study period, particularly during years marked by epidemiological outbreaks. Some of the interventions commonly implemented include risk communication, information on eliminating potential habitats such as empty containers and tires, cleaning pools and tanks, using repellent, wearing long-sleeved shirts, and using bed nets at night, along with neighborhood fumigation. In 2019, the World Mosquito Program (WMP) introduced its Wolbachia method in the city of Cali [41]. This involved releasing mosquitoes carrying Wolbachia, a bacterium that competes with other viruses such as dengue, Zika, chikungunya, and yellow fever, thus impeding the viruses’ ability to reproduce within the *Aedes aegypti* mosquito population. Future studies with longer time frames should consider the impact of this program in their modeling.

Regarding the limitations of this study, it is important to note that potential changes in epidemiological surveillance systems in various municipalities over a 19-year period could lead to imprecise data in terms of reported cases. This could bias the estimation of disease incidence patterns. Additionally, although we worked with confirmed cases, these are not always confirmed through laboratory testing (around 50% were confirmed) but rather through epidemiological linkage [42]. However, it is important to take into account that the database used is publicly available from the National Institute of Health and has already undergone review and cleaning processes [16]. Another limitation is the adjacency matrix used for the spatial random effect, which considers adjacent municipalities as neighbors. Some municipalities, such as Buenaventura on the Pacific coast or Cali, which have the largest populations and the greatest concentration of health services, showed different behaviors from the rest. This indicates that adjacent municipalities do not necessarily present the highest correlation. For future work, the construction of adjacency matrices should be explored, not necessarily defined by administrative boundaries. It is also important to consider that the validation process applied to the temperature series from satellite images can introduce bias, increasing the temperatures in some municipalities, as the gold standard method relied on a single station to represent the entire region.

This model allows us to make inferences and explain the effects of climate on dengue rates. However, while it successfully detects years with dengue epidemics, it overestimates the rates in some other years, indicating that it is not complex enough for accurate prediction. We plan to explore other methods, such as machine learning techniques, to construct a predictive model [43].

In conclusion, this study quantifies the non-linear and delayed relationships between dengue risk and climatic and socio-economic variables considering spatial and temporal variability in the Valle del Cauca department of Colombia. These findings could be valuable for local health authorities interested in developing early warning systems to predict future risks in advance. This is particularly important given the increasing frequency and intensity of extreme climate events due to climate change, and reinforces the need for robust models that can account the complexity of these phenomena.

## Supporting information

S1 Fig Mean temperature series. Comparison of time series from local station and satellite images.
(TIF)

S2 Fig Proportion of residents with access to water network. Basemap shapefile downloaded from https://rspatialdata.github.io/admin_boundaries.html.
(TIF)

S3 Fig Maximum temperature (°*C*) by municipality 2000–2019. (TIF)

S4 Fig Precipitation (mm) by municipality 2000–2019. (TIF)

S5 Fig Relative humidity (%) by municipality 2000–2019. (TIF)

S6 Fig Posterior predictive mean dengue rate 2001–2019. Basemap shapefile downloaded from https://rspatialdata.github.io/admin_boundaries.html.
(TIF)

S1 Table Correlation and linear regression models. Models between satellite images data and local station.
(DOCX)

## References

[1] World Health Organization. Dengue—Global situation 2023. Available at: https://www.who.int/emergencies/disease-outbreak-news/item/2023-DON498

[2] Organización Panamericana de la Salud. Informe de la situación epidemiológica del dengue en las Américas. 2023. Available at: https://www.paho.org/es/documentos/informe-situacion-no-3-situacion-epidemiologica-dengue-americas-semana-epidemiologica-02

[3] Morgan J., Strode C., & Salcedo-Sora J. E. Climatic and socio-economic factors supporting the co-circulation of dengue, Zika and chikungunya in three different ecosystems in Colombia. PLoS Neglected Tropical Diseases. 2021;15(3):e0009259. doi: 10.1371/journal.pntd.0009259 33705409 PMC7987142

[4] Carabali M., Harper S., Lima Neto A. S., dos Santos de Sousa G., Caprara A., Restrepo B. N., et al. Decomposition of socioeconomic inequalities in arboviral diseases in Brazil and Colombia (2007–2017). Transactions of The Royal Society of Tropical Medicine and Hygiene. 2022;116(8):717–726. doi: 10.1093/trstmh/trac004 35088864

[5] Ordonez-Sierra G., Sarmiento-Senior D., Gomez J. F. J., Giraldo P., Ramírez A. P., & Olano V. A. Multilevel analysis of social, climatic and entomological factors that influenced dengue occurrence in three municipalities in Colombia. One Health. 2021;12, 100234. doi: 10.1016/j.onehlt.2021.100234 33855157 PMC8025047

[6] Pavani J., Bastos L. S., & Moraga P. Joint spatial modeling of the risks of co-circulating mosquito-borne diseases in ceará, brazil. Spatial and Spatio-temporal Epidemiology. 2023; 47, 100616. doi: 10.1016/j.sste.2023.100616 38042535

[7] Farinelli E. C., Baquero O. S., Stephan C.,& Chiaravalloti-Neto F. Low socioeconomic condition and the risk of dengue fever: a direct relationship. Acta tropica. 2018; 180, 47–57. doi: 10.1016/j.actatropica.2018.01.005 29352990

[8] Aswi A., Cramb S. M., Moraga P., & Mengersen K. Bayesian spatial and spatio-temporal approaches to modelling dengue fever: a systematic review. Epidemiology & Infection. 2019, 147, e33. doi: 10.1017/S0950268818002807 PMC6518570 30369335

[9] Kirk D., Straus S., Childs M. L., Harris M., Couper L., Davies T. J., et al. A Temperature impacts on dengue incidence are nonlinear and mediated by climatic and socioeconomic factors: A meta-analysis. PLOS Climate. 2024; 3(3), e0000152. doi: 10.1371/journal.pclm.0000152

[10] Eastin M. D., Delmelle E., Casas I., Wexler J., & Self C. Intra-and interseasonal autoregressive prediction of dengue outbreaks using local weather and regional climate for a tropical environment in Colombia. American journal of tropical medicine and hygiene. 2014;91(3), 598. doi: 10.4269/ajtmh.13-0303 24957546 PMC4155567

[11] Yang H., Macoris M., Galvani K., Andrighetti M., & Wanderley D. Assessing the effects of temperature on the population of Aedes aegypti, the vector of dengue. Epidemiology & Infection. 2009; 137(8), 1188–1202. doi: 10.1017/S0950268809002040 19192322

[12] Azil A. H., Long S. A., Ritchie S. A., & Williams C. R. The development of predictive tools for pre‐emptive dengue vector control: a study of Aedes aegypti abundance and meteorological variables in North Queensland, Australia. Tropical Medicine & International Health. 2010; 15(10), 1190–1197. doi: 10.1111/j.1365-3156.2010.02592.x 20636303

[13] Ellis A. M., Garcia A. J., Focks D. A., Morrison A. C., & Scott T. W. Parameterization and sensitivity analysis of a complex simulation model for mosquito population dynamics, dengue transmission, and their control. The American journal of tropical medicine and hygiene. 2011; 85(2), 257. doi: 10.4269/ajtmh.2011.10-0516 21813844 PMC3144822

[14] Azil A. H., Long S. A., Ritchie S. A., & Williams C. R. The development of predictive tools for pre‐emptive dengue vector control: a study of Aedes aegypti abundance and meteorological variables in North Queensland, Australia. The American journal of tropical medicine and hygiene. 2010. 94(5), 1065. 20636303 10.1111/j.1365-3156.2010.02592.x

[15] Rodríguez R. C., Carrasquilla G., Porras A., Galera-Gelvez K., Yescas J. G. L., & Rueda-Gallardo J. The burden of dengue and the financial cost to Colombia, 2010–2012. Tropical Medicine & International Health. 2016, 94(5), 1065. 10.4269/ajtmh.15-0280 PMC4856604 26928834

[16] Instituto Nacional de Salud, Colombia. Informe de evento—Dengue. Available at: https://www.ins.gov.co/buscador-eventos/Paginas/Info-Evento.aspx

[17] Lowe R., Lee S. A., O’Reilly K. M., Brady O. J., Bastos L., Carrasco-Escobar G., et al. Combined effects of hydrometeorological hazards and urbanisation on dengue risk in Brazil: a spatiotemporal modelling study. The Lancet Planetary Health. 2021; 5(4), e209–e219. doi: 10.1016/S2542-5196(20)30292-8 33838736

[18] Desjardins M. R., Eastin M. D., Paul R., Casas I., & Delmelle E. M. Space–Time Conditional Autoregressive Modeling to Estimate Neighborhood-Level Risks for Dengue Fever in Cali, Colombia. The American Journal of Tropical Medicine and Hygiene. 2020; 103(5), 2040. doi: 10.4269/ajtmh.20-0080 32876013 PMC7646775

[19] Muñoz E., Poveda G., Arbeláez M. P., & Vélez I. D. Spatiotemporal dynamics of dengue in Colombia in relation to the combined effects of local climate and ENSO. Acta Tropica. 2021; 224, 106136. doi: 10.1016/j.actatropica.2021.106136 34555353

[20] Instituto Nacional de Salud (INS) Lineamientos para la vigilancia de Dengue en Colombia. Available at: https://www.ins.gov.co/buscador-eventos/Lineamientos/Pro_Dengue.pdf

[21] Departamento Administrativo Nacioanl de Estadística. Censo nacional de población y vivienda, Colombia 2018. Available at: https://microdatos.dane.gov.co/index.php/catalog/643/data-dictionary/F8?file_name=VIVENDAS

[22] Boogaard, H., Schubert, J., De Wit, A., Lazebnik, J., Hutjes, R., Van der Grijn, G. Agrometeorological indicators from 1979 to present derived from reanalysis. Copernicus Climate Change Service (C3S) Climate Data Store (CDS) 2020. Available at: https://cds.climate.copernicus.eu/cdsapp#!/dataset/10.24381/cds.6c68c9bb?tab=overview

[23] Reynolds, R.W., N.A. Rayner, T.M. Smith, D.C. Stokes, and W. Wang. An improved in situ and satellite SST analysis for climate 2002. Available at: https://psl.noaa.gov/data/timeseries/monthly/NINO12/

[24] Departamento Administrativo Nacional de Estadistica (DANE) Metodologia de la Estratificacion Socioeconomica Urbana para Servi- cios Publicos Domicioliarios. Direccion de Geoestadistica, ed. Santa Fe de Bogota, Colombia: DANE; 2015:96.

[25] Moraga P. Geospatial Health Data: Modeling and Visualization with R-INLA and Shiny. Chapman & Hall/CRC Biostatistics Series. 2019. https://www.paulamoraga.com/book-geospatial/

[26] Spiegelhalter D. J., Best N. G., Carlin B. P., & Van Der Linde A. Bayesian measures of model complexity and fit. Journal of the Royal Statistical Society Series B: Statistical Methodology. 2002; 64(4), 583–639. doi: 10.1111/1467-9868.00353

[27] Watanabe S., & Opper M. Asymptotic equivalence of Bayes cross validation and widely applicable information criterion in singular learning theory. Journal of machine learning research. 2010; 11(12).

[28] Held, L., Schrödle, B., & Rue, H. Posterior and cross-validatory predictive checks: a comparison of MCMC and INLA. Statistical modelling and regression structures: Festschrift in honour of ludwig fahrmeir. 2010; 91-110.

[29] Rue H., Martino S., & Chopin N. Approximate Bayesian inference for latent Gaussian models by using integrated nested Laplace approximations. Journal of the Royal Statistical Society Series B: Statistical Methodology. 2009; 71(2), 319–392. doi: 10.1111/j.1467-9868.2008.00700.x

[30] Moraga P. Small area disease risk estimation and visualization using R. The R Journal. 2018; 10(1), 495–506. https://journal.r-project.org/archive/2018/RJ-2018-036/index.html.

[31] Besag J., York J., & Mollié A. Small area disease risk estimation and visualization using R. Annals of the institute of statistical mathematics. 1991; 43, 1–20. doi: 10.1007/BF00116466

[32] Moraga P. Spatial Statistics for Data Science: Theory and Practice with R. Chapman & Hall/CRC Data Science Series. 2023. ISBN 9781032633510. https://www.paulamoraga.com/book-spatial/

[33] Lowe R., Gasparrini A., Van Meerbeeck C. J., Lippi C. A., Mahon R., Trotman A. R., et al. Nonlinear and delayed impacts of climate on dengue risk in Barbados: A modelling study. PLoS medicine. 2018; 15(7), e1002613. doi: 10.1371/journal.pmed.1002613 30016319 PMC6049902

[34] Gasparrini A. Modeling exposure–lag–response associations with distributed lag non‐linear models. Statistics in medicine. 2014; 33(5), 881–899. doi: 10.1002/sim.5963 24027094 PMC4098103

[35] Gasparrini A. Distributed lag linear and non-linear models in R: the package dlnm. Journal of Statistical Software. 2011; 43(8), 1–20. doi: 10.18637/jss.v043.i08 22003319 PMC3191524

[36] Organización Panamericana de la Salud. Dengue, guías para la atención de enfermos en la región de las Américas, 2015. Available at: http://iris.paho.org/xmlui/bitstream/handle/123456789/28232/9789275318904_esp.pdf?sequence=1&isAllowed=y

[37] Stewart Ibarra A. M., Ryan S. J., Beltrán E., Mejía R., Silva M., & Muñoz Á. Dengue vector dynamics (Aedes aegypti) influenced by climate and social factors in Ecuador: implications for targeted control. JPloS one. 2013; 8(11), e78263. doi: 10.1371/journal.pone.0078263 24324542 PMC3855798

[38] Hayden M. H., Uejio C. K., Walker K., Ramberg F., Moreno R., Rosales C., et al. Microclimate and human factors in the divergent ecology of Aedes aegypti along the Arizona, US/Sonora, MX border. EcoHealth. 2010; 7, 64–77. doi: 10.1007/s10393-010-0288-z 20232228

[39] Carabali M., Harper S., Lima Neto A. S., dos Santos de Sousa G., Caprara A., Restrepo B. N., et al. Spatiotemporal distribution and socioeconomic disparities of dengue, chikungunya and Zika in two Latin American cities from 2007 to 2017. Tropical Medicine & International Health. 2021, 26(3), 301–315. doi: 10.1111/tmi.13530 33219561

[40] Acosta, L.A. Evaluación de factores ambientales y climáticos como elementos de riesgo asociados con la transmisión de Dengue y la Leishmaniasis a sidferentes escalas temporales y espaciales en Colombia. Universidad Nacional de Colombia. 2015.

[41] World Mosquito Program. Wolbachia program. available at: https://www.worldmosquitoprogram.org

[42] Instituto Nacional de Salud (INS) Informe de evento dengue, Colombia, 2020. Available at: https://www.ins.gov.co/buscador-eventos/Informesdeevento/DENGUE_2020.pdf

[43] Mussumeci E., & Coelho F. C. Large-scale multivariate forecasting models for Dengue-LSTM versus random forest regression. Spatial and Spatio-temporal Epidemiology. 2020; 35, 100372. doi: 10.1016/j.sste.2020.100372 33138951

