# Supplementary figures and images for: Effects of climate variability on the spatio-temporal distribution of Dengue in Valle del Cauca, Colombia, from 2001 to 2019
Source: PLoS One. 2024 Oct 8;19(10):e0311607. doi: 10.1371/journal.pone.0311607 (PMC11460706; doi:10.1371/journal.pone.0311607)

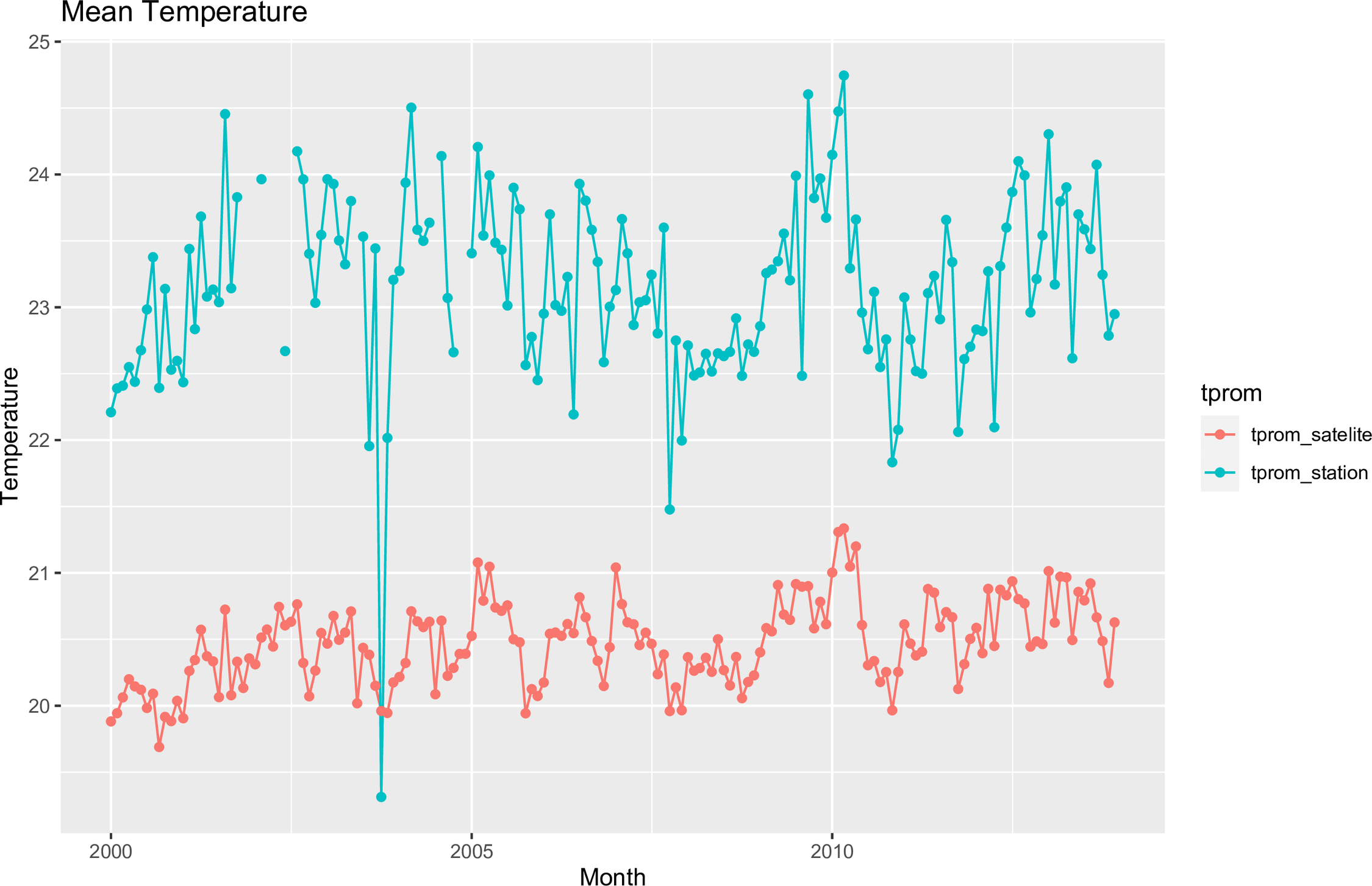

Supplement: S1 Fig — Comparison of time series from local station and satellite images. (TIF) [file pone.0311607.s001.tif]

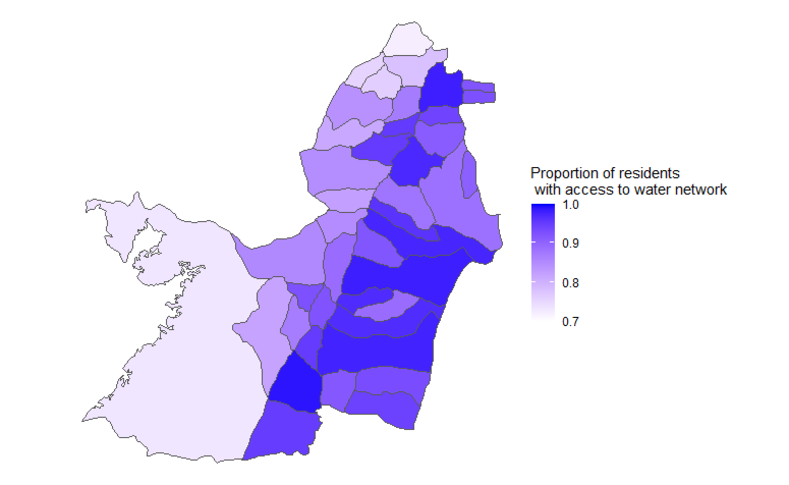

Supplement: S2 Fig — Basemap shapefile downloaded from https://rspatialdata.github.io/admin_boundaries.html. (TIF) [file pone.0311607.s002.tif]

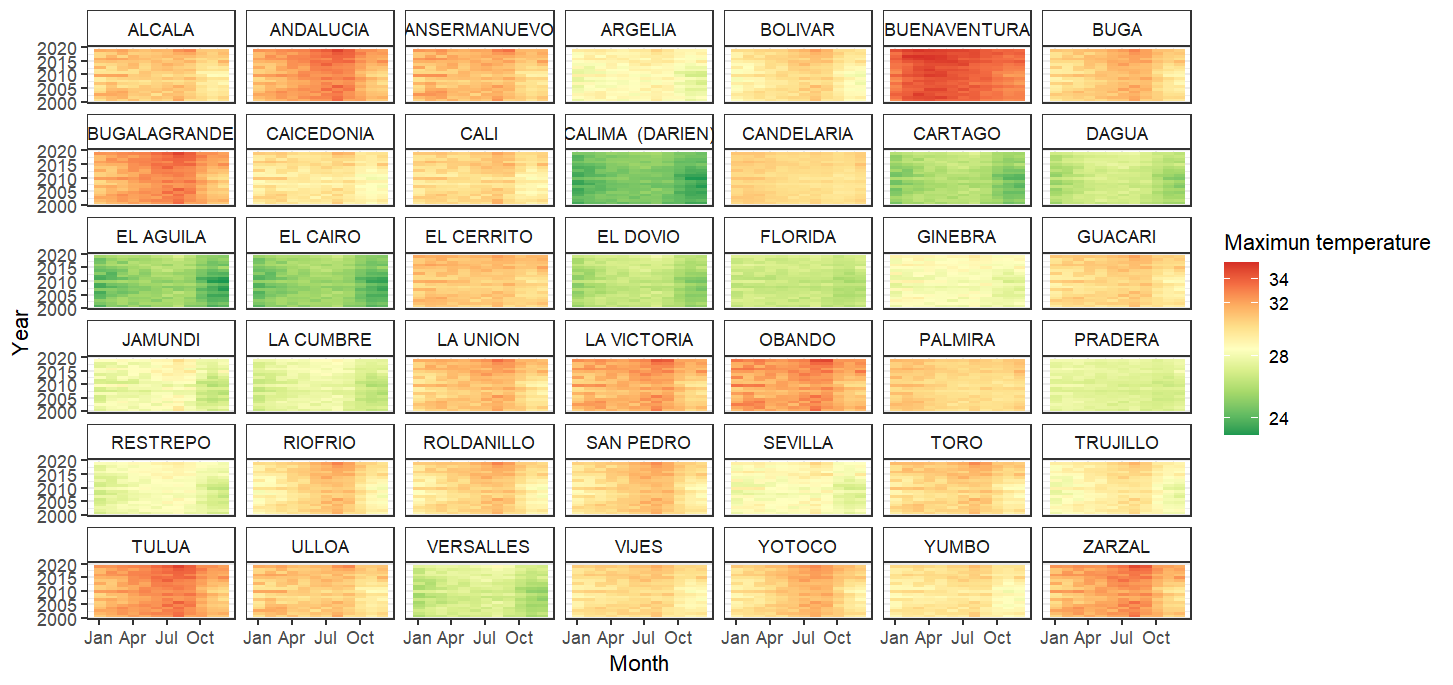

Supplement: S3 Fig — (TIF) [file pone.0311607.s003.tif]

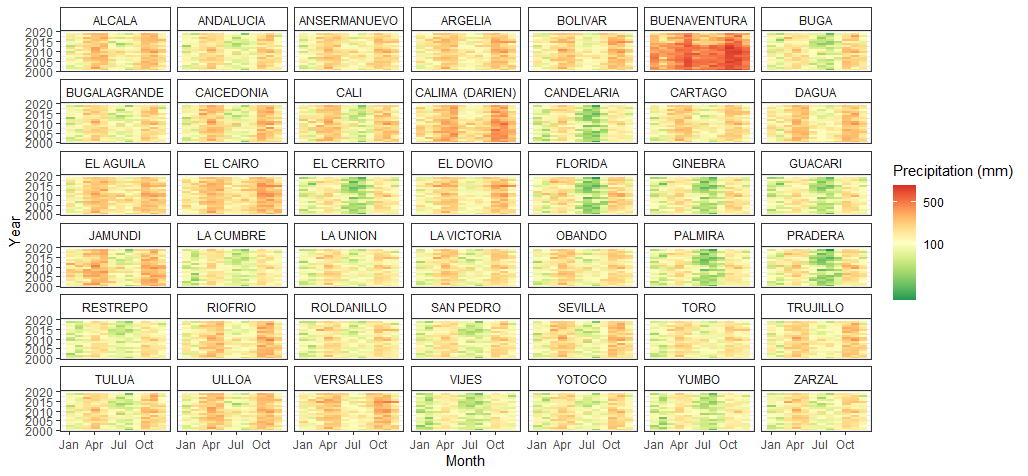

Supplement: S4 Fig — (TIF) [file pone.0311607.s004.tif]

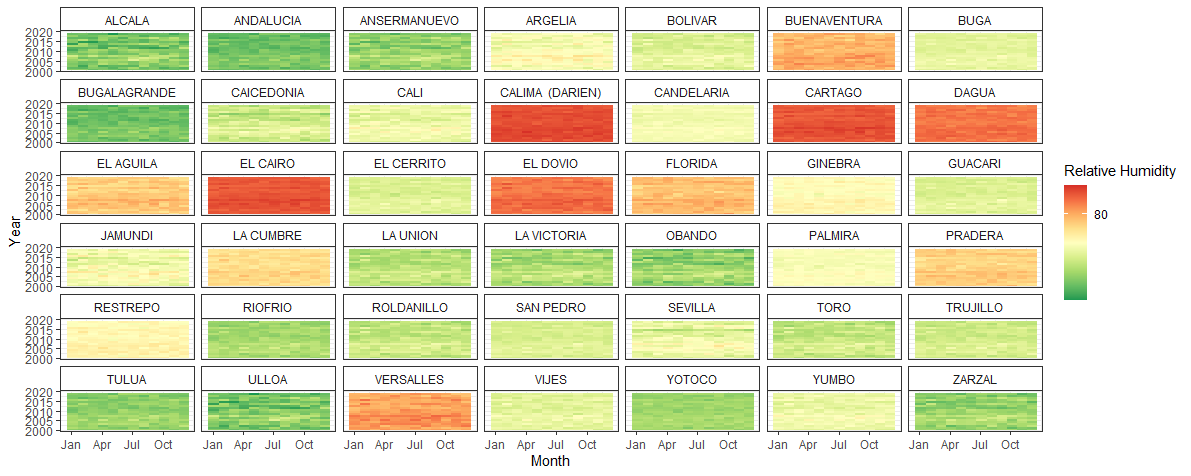

Supplement: S5 Fig — (TIF) [file pone.0311607.s005.tif]

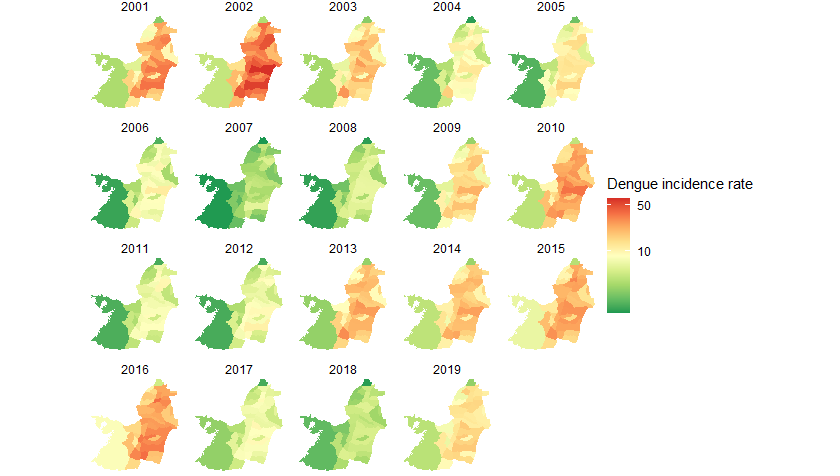

Supplement: S6 Fig — Basemap shapefile downloaded from https://rspatialdata.github.io/admin_boundaries.html. (TIF) [file pone.0311607.s006.tif]
