# Supplementary material for: Effects of climate variability on the spatio-temporal distribution of Dengue in Valle del Cauca, Colombia, from 2001 to 2019
Source: PLoS One. 2024 Oct 8;19(10):e0311607. doi: 10.1371/journal.pone.0311607 (PMC11460706; doi:10.1371/journal.pone.0311607)
Supplement: S1 Table — Models between satellite images data and local station. (DOCX) [file pone.0311607.s007.docx]

S1 Table. Linear regression models

| **Variable** | **Correlation** | **Intercept** | **Slope** |
| --- | --- | --- | --- |
| Maximun temperature | 0.70 | 1.65 | 1.16 |
| Minimun temperature | 0.68 | -13.43 | 1.81 |
| Mean  temperature | 0.66 | -6.33 | 1.44 |
